# The dynamics of MAPK inactivation at fertilization in mouse eggs

**DOI:** 10.1242/jcs.145045

**Published:** 2014-06-15

**Authors:** Jose Raul Gonzalez-Garcia, Josephine Bradley, Michail Nomikos, Laboni Paul, Zoltan Machaty, F. Anthony Lai, Karl Swann

**Affiliations:** 1 Institute of Molecular and Experimental Medicine, School of Medicine, Cardiff University, Cardiff CF14 4XN, UK; 2 School of Biosciences, Cardiff University, Cardiff CF10 3AX, UK; 3 Department of Animal Sciences, Purdue University, West Lafayette, IN 47907, USA

**Keywords:** Fertilization, MAPK, Egg, Zygote

## Abstract

Egg activation at fertilization in mammals is initiated by prolonged Ca^2+^ oscillations that trigger the completion of meiosis and formation of pronuclei. A fall in mitogen-activated protein kinase (MAPK) activity is essential for pronuclear formation, but the precise timing and mechanism of decline are unknown. Here, we have measured the dynamics of MAPK pathway inactivation during fertilization of mouse eggs using novel chemiluminescent MAPK activity reporters. This reveals that the MAPK activity decrease begins during the Ca^2+^ oscillations, but MAPK does not completely inactivate until after pronuclear formation. The MAPKs present in eggs are Mos, MAP2K1 and MAP2K2 (MEK1 and MEK2, respectively) and MAPK3 and MAPK1 (ERK1 and ERK2, respectively). Notably, the MAPK activity decline at fertilization is not explained by upstream destruction of Mos, because a decrease in the signal from a Mos–luciferase reporter is not associated with egg activation. Furthermore, Mos overexpression does not affect the timing of MAPK inactivation or pronuclear formation. However, the late decrease in MAPK could be rapidly reversed by the protein phosphatase inhibitor, okadaic acid. These data suggest that the completion of meiosis in mouse zygotes is driven by an increased phosphatase activity and not by a decline in Mos levels or MEK activity.

## INTRODUCTION

Before fertilization, mammalian oocytes are arrested at the second metaphase of meiosis. Upon fertilization, fusion with the sperm causes the egg to complete meiosis and the zygote to enter the first interphase of embryo development. The unfertilized egg is arrested in metaphase II as a result of the continuous activity of two main types of protein kinase (Carroll, 2001; Ducibella and Fissore, 2008). One of these is maturation-promoting factor (MPF), which consists of cyclin-dependent kinase 1 (CDK1) and cyclin B1 (Ducibella and Fissore, 2008; Madgwick and Jones, 2007). The other is a mitogen-activated protein kinase (MAPK) pathway, which comprises MAPK3 and MAPK1 (hereafter referred to as ERK1 and ERK2, respectively), which are stimulated by the sequential action of two upstream protein kinases, Mos and MAP2K1 and MAP2K2 (hereafter referred to as MEK1/2) (Verlhac et al., 1994; Verlhac et al., 1996; Dupré et al., 2011). Both MPF and the MAPK pathway become active during the final stages of oocyte maturation as the oocytes transition from arrest at the germinal vesicle stage through meiosis to arrest at meiotic metaphase II (Verlhac et al., 1994; Verlhac et al., 1996; Dupré et al., 2011). The increase in MAPK pathway activity is stimulated during maturation by increased synthesis of the upstream activator Mos. Exit from meiosis and entry into interphase at fertilization only occurs after both MPF and MAPK activities have undergone a large decrease (Verlhac et al., 1994; Moos et al., 1995; Moos et al., 1996). In frog, ascidian and mammalian eggs, the decline in MPF activity is known to be primarily due to the proteolysis of cyclin B, which is triggered by the activation of the protease activity of the anaphase-promoting complex (APC). The APC itself is stimulated by the destruction of Emi2 (Madgwick and Jones, 2007). Emi2 acts as an inhibitor of the APC and as a stabilizer of metaphase II, and is itself stabilized by Mos-stimulated MAPK activity (Suzuki et al., 2010; Dupré et al., 2011). The loss of Emi2 and cyclin B, and the subsequent decline in MPF activity can explain the earliest events of meiotic resumption, such as metaphase anaphase transition and the second polar body emission (Shoji et al., 2006; Suzuki et al., 2010). However, MAPK activity is high for longer than MPF activity in order to maintain spindle integrity in the events leading up to second polar body emission (Verlhac et al., 1994; Petrunewich et al., 2009). MAPK pathway inactivation appears to be delayed until around the time of pronuclear formation, which signals the end of egg activation and marks the entry into interphase of the first cell cycle (Verlhac et al., 1994; Marangos et al., 2003). Experiments using constitutively active MEK or phosphatase inhibitors, such as okadaic acid, have shown that there is an absolute requirement for a fall in MAPK activity before pronuclei are formed in mouse zygotes (Moos et al., 1995; Moos et al., 1996). However, other studies on artificially activated eggs suggest that MAPK dephosphorylation might not occur until after pronuclear formation, and so the role of a decline MAPK activity in this event has been questioned (Soeda et al., 2013). However, unlike MPF, the exact timing of MAPK inactivation has not been measured and there are no evident causal links between the signaling pathways triggered by the sperm and the delayed decline in MAPK activity.

The initial signal that the fertilizing sperm triggers in order to release the egg from metaphase II arrest in vertebrates is a large Ca^2+^ increase in the egg cytosol (Stricker, 1999; Ducibella and Fissore, 2008). In frogs, the sperm triggers a single Ca^2+^ increase, whereas in mammals sperm–egg fusion leads to a prolonged series of Ca^2+^ oscillations (Stricker, 1999). In mammalian eggs, there is substantial evidence suggesting that the sperm causes these Ca^2+^ oscillations by the introduction of a sperm-specific phospholipase C, PLCζ, after gamete membrane fusion (Saunders et al., 2002). PLCζ then hydrolyzes its substrate phosphatidylinositol (4,5)-bisphosphate (PIP_2_) to drive repetitive cycles of inositol trisphosphate (IP_3_) production and Ca^2+^ release that last for several hours (Swann and Lai, 2013). In mouse eggs, the Ca^2+^ oscillations eventually stop at the time of pronuclear formation (Marangos et al., 2003; Ito et al., 2008) owing to the sequestration of PLCζ within the nascent pronuclei (Larman et al., 2004). In both frogs and mammals, the Ca^2+^ signal stimulates Ca^2+^/calmodulin-dependent protein kinase II (CaMKII), which leads to phosphorylation of Emi2 and eventually results in Emi2 proteolysis (Madgwick and Jones, 2007). Hence, the Ca^2+^ signals have been linked to the loss of MPF activity (Madgwick and Jones, 2007; Suzuki et al., 2010). In contrast, the decline in MAPK activity occurs several hours later and has not been linked to Ca^2+^ signals (Verlhac et al., 1994; Marangos et al., 2003). Because the activation of the MAPK pathway comprises the protein kinase cascade of Mos, MEK and ERK1/2, it is possible that the late fall in MAPK could be linked to a destruction of Mos, and then a loss of MEK activity (Dupré et al., 2011). This model is supported in ascidian oocytes, where the overexpression of Mos prevents the decline in MAPK and blocks pronuclear formation (Dumollard et al., 2011). Also in support of this idea, Mos protein in mouse eggs has been shown to decline at some time between second polar body emission and pronuclear formation during parthenogenetic activation (Weber et al., 1991). However, whether this decline in Mos levels correlates with, or is required for, a decline in MAPK activity and pronuclear formation is unclear. Furthermore, Mos appears to be unstable in mouse eggs because Mos protein levels also decline over several hours in unfertilized eggs (Shoji et al., 2006). Hence, the presumed link between the loss of Mos and the fall in MAPK activity in mammalian eggs has remained unresolved.

The timing of cyclin B destruction at fertilization in mouse eggs has been monitored in real time by imaging the loss of either GFP- or luciferase-tagged fusion proteins (Nixon et al., 2002; Marangos and Carroll, 2004; Ajduk et al., 2008). The decline in cyclin B levels can be an effective surrogate for the fall in MPF activity, and its decline starts during the initial Ca^2+^ rise. The fall in MPF is then completed within the next two or three Ca^2+^ spikes, which are within 20 min of the initial Ca^2+^ increase (Nixon et al., 2002; Marangos and Carroll, 2004; Ajduk et al., 2008). Despite the significance of MAPK in cell cycle transitions in eggs, there are no comparable dynamic imaging studies on cell cycle changes in Mos levels or MAPK activity. This might be primarily because Mos protein is present at very low (∼nM) levels in eggs, which is below the typical detection limit of fluorescent proteins (Ferrell, 1996; Niswender et al., 1995). Furthermore, the changes in MAPK activity are caused by a dephosphorylation of ERK1 and not its proteolysis, and so the relevant change in MAPK can only be detected using groups of eggs combined with either *in vitro* kinase assays or western blots using antibodies against phosphorylated proteins (Verlhac et al., 1994; Moos et al., 1995). In this study, we have applied a split-luciferase-based probe to monitor the activity of the MAPK pathway. This activity indicator reports the conformational change in ERK1 that occurs upon phosphorylation by MEK (Khokhlatchev et al., 1998; Cobb and Goldsmith, 2000). This chemiluminescent indicator has been used to demonstrate the precise timing of decrease in ERK1 phosphorylation in relation to the sequence of Ca^2+^ oscillations during fertilization of mouse eggs. The data suggest that MAPK activity starts to decrease several spikes prior to the cessation of the series of Ca^2+^ transients but that the complete inactivation of MAPK does not occur until after pronuclei have formed. We have also examined changes in recombinant Mos in fertilizing mouse eggs using a Mos–luciferase fusion protein, which can be detected at low expression levels. We show that there is no measurable increase in Mos–luciferase destruction at fertilization, and that overexpression of Mos does not affect the timing of MAPK activity decrease or the formation or pronuclei. By contrast, our data show that the fall in MAPK pathway activity during the final stages of egg activation is driven by an increase in okadaic-acid-sensitive phosphatase activity.

## RESULTS

### Monitoring MAPK activity with the ERK1 split-luciferase reporter MAPK_AR_

**Fig. 1.**
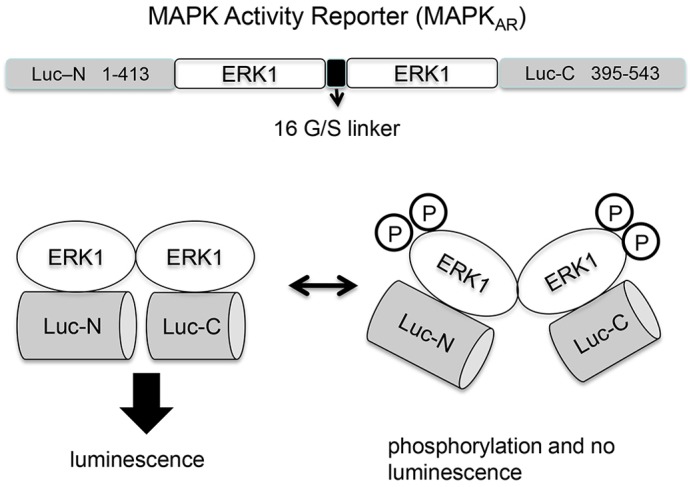
**Schematic illustration of the MAPK_AR_.** Two tandem ERK1 monomers were inserted between the N-terminal and C-terminal fragments of click beetle luciferase, and coupled by a flexible glycine-serine (G/S) linker. MAPK activation (phosphorylation and ERK1 interaction) keeps the split-luciferase halves apart and no emission of luminescence occurs. MAPK inactivation triggers a conformational change that brings the split luciferase halves together and a luminescent signal is emitted.

**Fig. 2.**
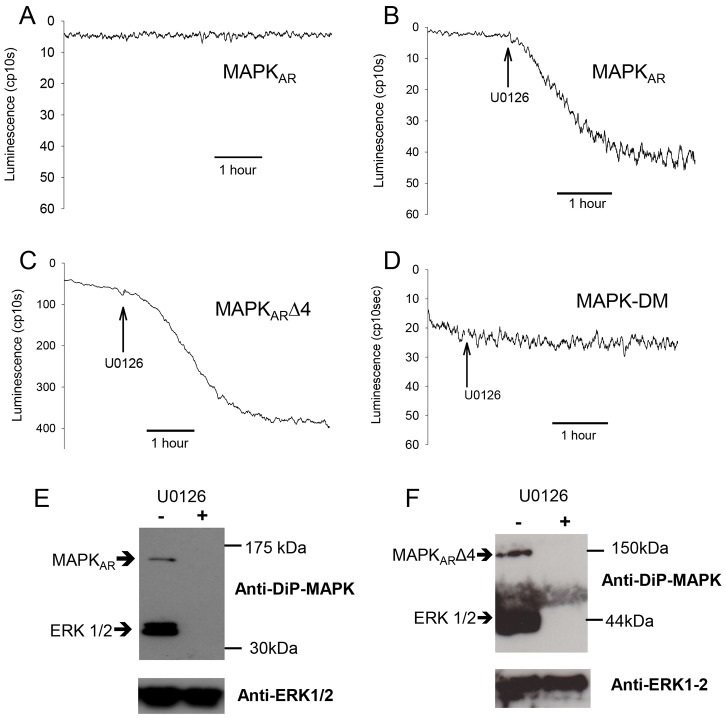
**The response of MAPK_AR_ and its variants to U0126.** The luminescence recordings (in counts per 10 seconds) are shown from sample eggs previously injected with cRNA encoding for each probe and incubated in 100 µM luciferin. (A) The egg was left for several hours without addition of drugs (one trace is shown that is representative of 10 eggs). (B) 50 µM of the MEK inhibitor U0126 was added to an egg expressing MAPK_AR_ at the time indicated by the arrow, which leads to an increase in luminescence (one trace is shown that is representative of 26 eggs). (C) 50 µM U0126 was added to an egg expressing MAPK_AR_Δ4 (one trace is shown that is representative of 53 eggs), and (D) 50 µM of U0126 was added to an egg expressing MAPK-DM (one trace is shown that is representative of 27 eggs). (E,F) Western blots using antibodies against diphosphorylated ERK1/2 and ERK1/2. Eggs (groups of 50–60) were injected with either MAPK_AR_ (E) or MAPK_AR_Δ4 (F) cRNA and incubated for 3–4 h. In E, the first lane is from eggs expressing MAPK_AR_, and the second lane from eggs that were treated with 50 µM U0126 for the last hour before lysis in sample buffer. The upper blot is with antibody against diphosphorylated (DiP) ERK1/2, and the lower blot with anti-ERK1/2 antibodies. In F the conditions are the same as in E with control eggs and those treated with 50 µM U0126, except that the eggs were expressing the MAPK_AR_Δ4 probe for both lanes.

In order to measure changes in MAPK activity within intact mouse eggs, we designed a genetically-encoded bioluminescent reporter that is capable of monitoring the activity of the terminal protein kinase in the MAPK pathway, namely ERK. This new luciferase reporter is a single molecule that consists of two tandem ERK1 sequences inserted between the N- and C-terminal portions of click beetle luciferase and is referred to as a MAPK activity reporter or MAPK_AR_. Fig. 1 is a schematic diagram of the design of the reporter. Fig. 2A shows the luminescence from unfertilized mouse eggs injected with cRNA encoding the MAPK_AR_. The luminescent signal was maintained at a low level in unfertilized eggs, but it increased substantially after addition of the MEK inhibitor U0126 (Fig. 2B). U0126 has been shown to be a specific inhibitor of MEK in mouse eggs and its application leads to the dephosphorylation of ERK1/2 in mouse eggs (Phillips et al., 2002). Consequently, the data suggest that MAPK_AR_ undergoes a conformational change that causes an increase in luminescence when ERK1 dephosphorylation and inactivation occurs (Fig. 1). Thus, a decreased interaction between the two ERK1 moieties within MAPK_AR_ enables structural and functional reassociation of the N- and C-terminal domains of click beetle luciferase to reconstitute bioluminescence activity, as has been proposed for the firefly split-luciferase reporter of Akt kinase activity (Zhang et al., 2007). In order to make the interpretation of traces more intuitive, note that the luminescent recordings are inverted so that the inactivation of ERK1 corresponds to a downward deflection in the trace. From Fig. 2B it is evident that the luminescence signal change upon U0126 addition is about ten-fold, given that starting luminescence values were 0.1–0.5 counts per second (cps) and after U0126 treatment the signal increases were typically 4–5 cps.

Previous studies have shown that ERK1/2 monomers undergo dimerization upon phosphorylation (Cobb and Goldsmith, 2000). We also generated a related chemiluminescent reporter based upon an altered version of ERK1 that cannot dimerize owing to the deletion of four residues (Lidke et al., 2010). This interaction-deficient ERK1 reporter, which was designed and constructed as for MAPK_AR_, is referred to as MAPK_AR_Δ4. When MAPK_AR_Δ4 was injected into eggs, it showed a higher basal expression level (luminescence >3 cps) compared to that of MAPK_AR_ (Fig. 2C). Upon addition of U0126, the MAPK_AR_Δ4-injected eggs showed a similar order of magnitude change in luminescence as found for MAPK_AR_ (Fig. 2B). The higher basal luminescence for MAPK_AR_Δ4 indicates interaction between the N- and C-terminal luciferase fragments likely occurs owing to the defective interaction between the ERK1 moieties. However, because MAPK_AR_Δ4 shows a large change in luminescence after U0126 treatment, it suggests that, after dephosphorylation, there is further considerable movement of the interaction-deficient ERK1 subunits.

In order to establish that the above luminescence signal changes are dependent upon phosphorylation, a further ‘MAPK reporter’ lacking the two phosphorylation sites (T202, Y204) in each of the ERK1 monomer sequences was produced (Cobb and Goldsmith, 2000). This related construct to those described above, is referred to as the ERK1 phosphorylation double mutant (MAPK-DM). Unlike the other MAPK_AR_, MAPK-DM cannot be phosphorylated in the unfertilized egg. When the cRNA for MAPK-DM was injected into mouse eggs there was a clear luminescence signal in unfertilized eggs in the absence of any stimuli (Fig. 2D). The subsequent addition of U0126 did not have any effect on the luminescence signal in any of the eggs recorded. This suggests that the U0126-mediated luminescence changes seen with the MAPK_AR_ and MAPK_AR_Δ4 proteins require specific phosphorylation of the T202 and Y204 residues (Fig. 2D).

The luminescence changes in MAPK_AR_-injected eggs treated with U0126 were next correlated with the dephosphorylation patterns of both the endogenous ERK1 and recombinant MAPK_AR_. The immunoblots in Fig. 2E show that phosphorylation of ERK1 in untreated eggs and its dephosphorylation following U0126 addition both exhibited a pattern that is entirely consistent with the luminescence recordings. Immunoblot analysis further indicated that the expressed MAPK_AR_Δ4 was also dephosphorylated at the same time as the endogenous ERK1 in mouse eggs exposed to U0126 (Fig. 2F). We note from these immunoblots that the MAPK_AR_ probes were expressed at much lower concentrations than the endogenous ERK1/2. The longer exposures used to reveal the MAPK_AR_ probes meant there was poor resolution of separate ERK1 and ERK2 bands that can otherwise be seen in supplementary material Fig. S1. Taken together, these results show that the luminescence signal changes derived from these various MAPK_AR_ expression constructs appear to faithfully report ERK1 phosphorylation and hence activation of the MAPK pathway in mouse eggs. In view of their place in the MAPK pathway (downstream of MEK), the MAPK_AR_ luminescent probes can be regarded as surrogate reporters for the state of ERK1 phosphorylation and, hence, the degree of activation of MEK, the penultimate kinase in the MAPK pathway.

### MAPK dynamics during fertilization

**Fig. 3.**
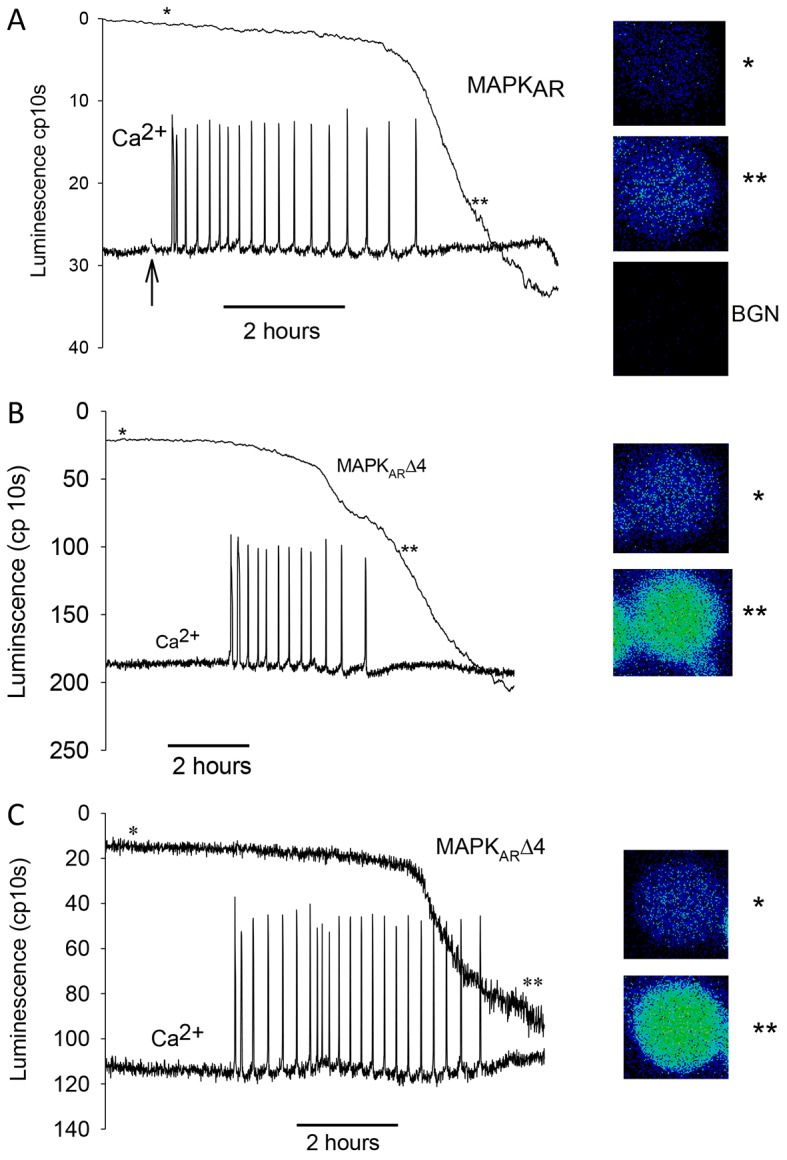
**MAPK changes during fertilization.** (A) An egg expressing MAPK_AR_ and injected with OGBD was fertilized and its luminescence recorded alongside Ca^2+^ oscillations (the fluorescence of OGBD dye measuring Ca^2+^ are in arbitrary units). The change in luminescence towards the end of the series of Ca^2+^ oscillations is typical of 41 eggs. (B) A similar experiment to A, except that the MAPK_AR_Δ4 probe was used, and the recording shown is typical of 23 eggs. (C) Another recording from a MAPK_AR_Δ4-injected egg where the luminescence trace was not filtered, in order to illustrate the nature of the start of the change in MAPK_AR_Δ4 luminescence during fertilization. The images on the right-hand sides of each set of traces show the luminescence of egg for 10 min before and after fertilization (at the times in the traces indicated by * and **, ‘BGN’ shows the background count). In A, sperm were added to the recording dish at the point indicated by the arrow, in B and C sperm were added before the start of the trace.

Using MAPK_AR_ and MAPK_AR_Δ4, we then examined the timing of the decrease in MAPK activity during fertilization. We carried out experiments where the luminescence of our MAPK probes was measured alongside the fluorescence of a Ca^2+^-sensitive dye. Fig. 3A shows a typical recording from mouse eggs during fertilization, simultaneously measuring both Ca^2+^ oscillations and the luminescence signal changes from MAPK_AR_. Images of the luminescent eggs before and after fertilization are shown in the insets in Fig. 3A–C. In all experiments, the luminescence was low before fertilization and during the early phase of the Ca^2+^ transients. After a couple of hours, there was a change in luminescence that started before, but was not completed until well after, the cessation of Ca^2+^ oscillations. In fertilized eggs where we monitored the full series of Ca^2+^ oscillations and change in MAPK_AR_ signal, the time period from the first Ca^2+^ increase to the cessation of Ca^2+^ oscillations was 4 h 5 min (±10 min, s.e.m.), whereas the time that had elapsed between the first Ca^2+^ transient and the half maximal change in MAPK_AR_ luminescence was 6 h 38 min (±17 min, s.e.m.; 41 fertilized eggs from four separate experiments). The change in luminescence commenced at between 2 and 3 h after the initiation of Ca^2+^ oscillations. In every experiment where there was a detectable luminescence signal at the beginning of Ca^2+^ oscillations, the inflection point indicating the start of the change in MAPK_AR_ luminescence always occurred before the last of the series of Ca^2+^ oscillations. In many, but not all cases, there appeared to be two phases of luminescence change with one phase before and another after the cessation of Ca^2+^ oscillations.

Fertilization experiments were also conducted using MAPK_AR_Δ4 (Fig. 3B). With this reporter there was a higher basal luminescence during the initial phase of the Ca^2+^ transients. As for MAPK_AR_, there was an even more distinct change in MAPK_AR_Δ4 luminescence observed towards the end of the series of Ca^2+^ oscillations that continued for several hours after the cessation of Ca^2+^ oscillations (Fig. 3B,C). The start of the change in signal for MAPK_AR_Δ4 was more evident because it showed a higher level of luminescence before a marked inflexion with the change in luminescence often appearing as a distinct switch (e.g. Fig. 3C). Eggs injected with MAPK_AR_Δ4 that did not fertilize did not show such an inflexion in the trace, nor did they show the marked increase in luminescence of fertilized eggs (see supplementary material Fig. S2). Owing to the higher initial luminescence values, we were able to estimate that the decline in MAPK_AR_Δ4 signal started 2 h 25 min (±12 min, *n* = 23, s.e.m.) after the first Ca^2+^ increase at fertilization. This time point corresponds to the occurrence of the 18th Ca^2+^ transient out of 25 Ca^2+^ transients (mean values) suggesting that the change in MAPK begins about three quarters of the way through the total number of Ca^2+^ transients. The fertilization-induced luminescence signal with MAPK_AR_Δ4 also showed signs of a two-phase change, with one phase before and another after the cessation of Ca^2+^ oscillations. It is known that the cessation of Ca^2+^ oscillations in mouse zygotes correlates with the time of pronuclear formation (Marangos et al., 2003). It was noteworthy that when we fertilized eggs injected with the MAPK-DM, which is a version of our probes that cannot be phosphorylated, there was no distinct change, or distinct inflexion in the trace, as seen with the MAPK_AR_ and MAPK_AR_Δ4 probes (supplementary material Fig. S3.) These data show that the changes in luminescence we see with the MAPK_AR_ and MAPK_AR_Δ4 probes reflect phosphorylation of ERK1. Consequently, our data with two different ERK1-based reporters suggest that the onset of MAPK inactivation starts abruptly towards the end of the series Ca^2+^ oscillations at fertilization, a point well before pronuclear formation. However, the MAPK inactivation then continues progressively for a considerable time after pronuclear formation.

### The role of Mos in MAPK inactivation and pronuclear formation

**Fig. 4.**
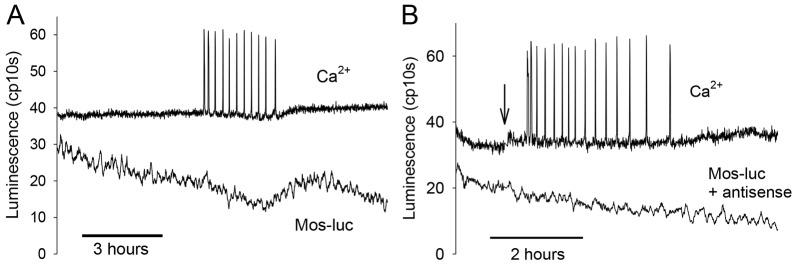
**Changes in Mos–luc luminescence during fertilization.** Eggs were injected with Mos–luc cRNA and OGBD and allowed to express Mos–luc before fertilization. (A) An example of an egg (typical of 19 eggs) that was fertilized, as indicated by the presence of Ca^2+^ oscillations. The Mos–luc luminescence is shown in the lower trace. (B) An example of a representative egg injected sequentially with Mos–luc RNA and antisense Mos–luc (3 h later), and OGBD before the start of the recording. Sperm were added (at the arrow in B and before the start of the trace in A) and eggs started fertilization at the time marked by the Ca^2+^ oscillations. The Mos–luc luminescence is shown on the lower trace (one of 29 similar recordings).

The delayed decrease in MAPK activity at fertilization could be due to delayed destruction of Mos protein. We therefore investigated the stability of Mos during fertilization by making a Mos–luciferase (Mos–luc) fusion protein, in a similar approach to previous studies of cyclin B stability (Nixon et al., 2002; Ajduk et al., 2006). Fig. 4A shows the luminescence recorded from a fertilizing egg expressing Mos–luc, as well as the Ca^2+^ oscillations measured with a fluorescent dye. Because Mos is considered to be present at a very low concentration in eggs, we injected small amounts of Mos–luc cRNA to produce a low luminescence signal consistent with the level of reporter expression (see Discussion). Fig. 4A shows that there was a steady decline in Mos–luc luminescence before the egg was fertilized. During the series of Ca^2+^ oscillations produced at fertilization the luminescence decline continued at the same rate. However, towards the end of the Ca^2+^ oscillations there was a distinct increase in Mos–luc luminescence seen in all fertilized eggs (Fig. 4A). The increase in Mos–luc luminescence late in fertilization likely arises from the synthesis of Mos–luc protein from the injected cRNA exceeding the rate of protein destruction. We could prevent this increase by injecting eggs sequentially with Mos–luc cRNA, and then 3 h later, with Mos–luc antisense RNA to inhibit further Mos–luc expression. In this case, the distinct increase was absent and a steady decline in Mos–luc luminescence was maintained during fertilization (Fig. 4B). These data, using two different protocols, suggest that Mos does not undergo increased destruction during fertilization-induced Ca^2+^ oscillations in mouse eggs.

**Fig. 5.**
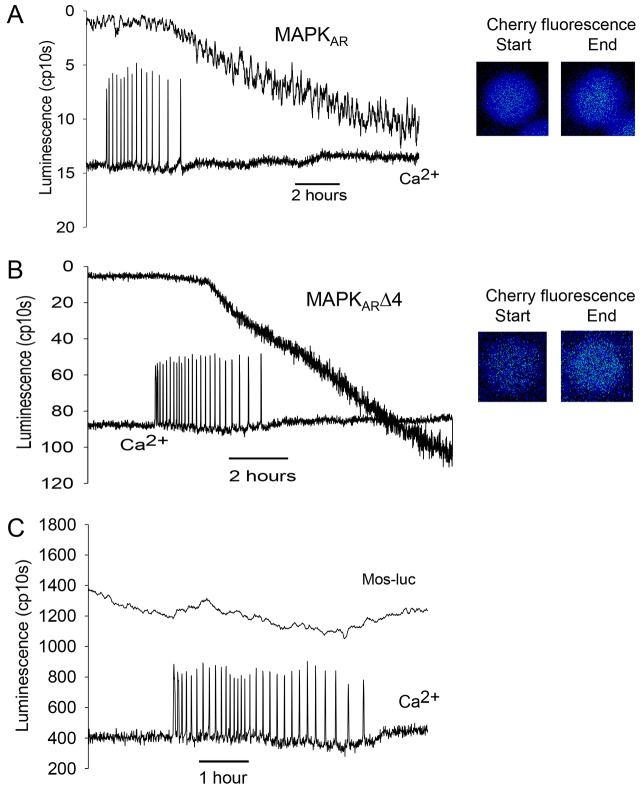
**Mos overexpression and fertilization.** (A,B) Traces from an egg injected with MAPK_AR_ and Mos-Cherry cRNA ∼4 h before the start of the recording. The sperm were added before the start of the traces shown. The fluorescence of Mos–Cherry was measured just before recording, and then the fluorescence of OGBD and the luminescence of MAPK_AR_ (A) and MAPK_AR_Δ4 (B) were recorded during fertilization as in Fig. 3. In experiments of which A is an example, the mean Mos–Cherry signal was 7.3 times the background fluorescence (*n* = 12). In experiments of which B is an example, the mean Mos–Cherry signal of the egg was 6.6 times the background fluorescence (*n* = 16). In each case, the pattern of luminescence change was similar to control eggs that had not been injected with Mos–mCherry cRNA. The images show the fluorescence of Mos–Cherry in the same eggs as shown in the traces for the start and end of the experiment. (C) A recording from one of 30 eggs that were injected with Mos–luc cRNA and Ca^2+^ dye ∼3 h before the start of the recording. Conditions were otherwise the same as Fig. 3.

We next wanted to investigate whether overexpression of Mos would perturb the temporal profile of the MAPK decrease or the formation of pronuclei. Mos overexpression has previously been shown to block MAPK inactivation in fertilizing ascidian eggs, and this response is seen with Mos from a variety of species (Dumollard et al., 2011). We were not able to monitor MAPK_AR_ alongside Mos–luc because they both generate the same luminescent signal, so we used the same Mos–Cherry construct that was used effectively in ascidian oocytes (Dumollard et al., 2011). Fig. 5A,B depicts the changes in the MAPK_AR_ or MAPK_AR_Δ4 signal in mouse egg co-injected with ascidian Mos–Cherry. In both instances, the change in luminescence occurred in a similar manner to that in control eggs and all such mouse eggs expressing Mos–Cherry also formed pronuclei. For eggs expressing MAPK_AR_Δ4 and Mos–Cherry, the time from the initial Ca^2+^ transient to the point at which MAPK started to decline was 1 h 54 min (±7 min; *n* = 20, s.e.m.). This is not significantly different to the 2 h 2 min (±5 min, *n* = 13, s.e.m.) observed for the corresponding time for control eggs not expressing Mos–Cherry, and which were simultaneously fertilized in the same experiment. The average level of Mos–Cherry fluorescence in all these experiments was ∼5 times greater than the egg autofluorescence. These data suggest that Mos expression in mouse eggs does not affect the onset or the subsequent time course of the decline in the MAPK pathway activity at fertilization.

We also examined whether the mouse Mos–luc itself could affect the time course of pronuclear formation because this depends upon a decline in MAPK levels (Moos et al., 1996). Because the first Ca^2+^ transients initiate the events of metaphase–anaphase transition, and the termination of Ca^2+^ oscillations is precisely correlated with pronuclei formation in mouse eggs, we again used the start and cessation of Ca^2+^ oscillations as a proxy for measuring the period required for eggs to form pronuclei and enter interphase. Fig. 5C shows an example of Ca^2+^ oscillations recorded from a fertilizing mouse egg injected with a high concentration of Mos–luc cRNA to induce luminescence expression levels ∼1000 times higher than in the above experiments (Fig. 5A,B). The Ca^2+^ oscillations in the presence of high Mos–luc luminescence displayed a similar pattern to control eggs. There is a slight decline in Mos–luc before fertilization, but no major loss of this signal during fertilization. Fig. 5C shows, in fact, that after Ca^2+^ oscillations start there is a slight increase in luminescence, probably due to Ca^2+^-induced stimulation of mitochondrial ATP production (Campbell and Swann, 2006). These Mos–luc-injected eggs all displayed Ca^2+^ oscillations and pronuclei formation in a manner similar to control eggs simultaneously fertilized in the same dish. The duration and number of Ca^2+^ oscillations was 3 h 53 min (±76 min, s.d.) with 46.5 (±17.7, s.d.) Ca^2+^ spikes for eggs with high levels of Mos–luc injected (all 30 eggs expressing >100 cps of Mos–luc), compared to 3 h 18 min (±43 min, s.d.) and 46.9 (±23.5, s.d.) Ca^2+^ spikes for 18 parallel control eggs. Consequently, these results indicate that overexpression of Mos–luc does not alter the timing of pronuclei formation. Combined with the above measurements of Mos–luc decline, our data suggest that increased Mos destruction does not occur in response to sperm-induced Ca^2+^ signals. In addition, the continued presence of Mos protein in eggs does not affect the timing of MAPK activity decrease, the occurrence and termination of Ca^2+^ oscillations, or the formation of pronuclei.

### The decline in MAPK activity, Ca^2+^ signals and pronuclear formation

**Fig. 6.**
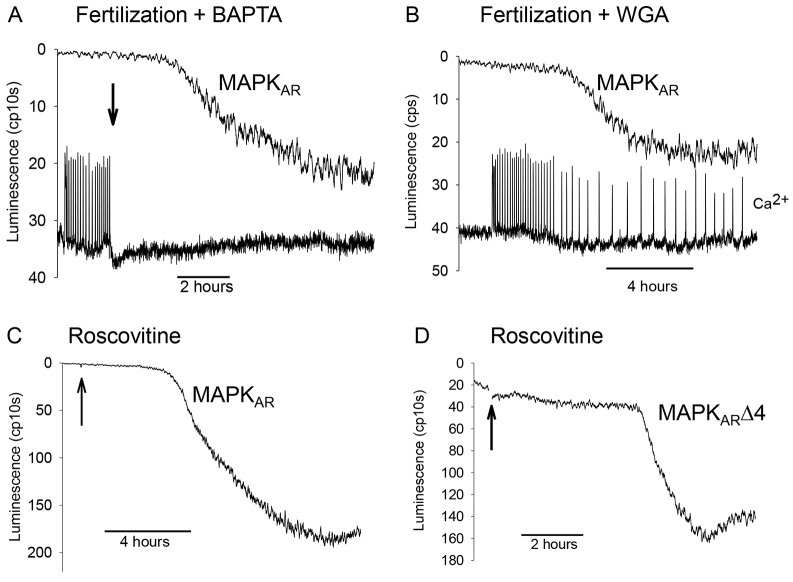
**The role of Ca^2+^ in the timing of the MAPK decrease.** (A) A representative recording from an egg injected with MAPK_AR_ and, once Ca^2+^ oscillations had started, BAPTA (final concentration of 2 mM) was added to the drop containing the eggs in order to terminate Ca^2+^ oscillations. The response is typical of 33 eggs where all eggs showed a change in MAPK_AR_ luminescence. (B) A representative trace from a single egg injected with WGA, as well as OGBD and MAPK_AR_ cRNA. The Ca^2+^ oscillations at fertilization lasted longer than usual and a similar change in MAPK_AR_ luminescence to the one shown was seen in all 29 eggs. In A and B, sperm were added to the dish before the start of traces. (C,D) Eggs expressing either MAPK_AR_ or MAPK_AR_Δ4 respectively were treated with 50 µM roscovitine (added at the point indicated by the arrow). Traces are representative of three eggs for C and 12 eggs for D. The conditions for C and D are as in Fig. 2.

Because the MAPK decline starts during the Ca^2+^ oscillations, we next determined whether the dynamics of MAPK decline was influenced by the presence or absence of Ca^2+^ signals. When Ca^2+^ oscillations are initiated in fertilized eggs, they can be stopped by addition of sufficient BAPTA to chelate extracellular Ca^2+^ (Fig. 6A), but notably, the change in MAPK_AR_ signal still occurred as in control eggs that displayed normal Ca^2+^ oscillations. However, the time to reach the half-maximal change in MAPK_AR_ luminescence was 8 h 26 min (±52 min, s.e.m.) for BAPTA-treated eggs (*n* = 33) which is slightly longer than the mean time for control eggs reported above (6 h 38 min; ±17 min, s.e.m.). This data suggests that whereas premature termination of Ca^2+^ oscillations still allows MAPK inactivation, the kinetics of inactivation are slightly delayed by the absence of Ca^2+^ signals.

The converse situation to stopping the fertilization-induced Ca^2+^ oscillations early can be achieved by inhibiting pronuclear formation. In wheat germ agglutinin (WGA)-injected mouse eggs, pronuclear formation is prevented and Ca^2+^ oscillations persist for a prolonged period owing to the absence of nuclear localization of PLCζ (Marangos et al., 2003; Larman et al., 2004). Fig. 6B shows a recording of the MAPK_AR_ signal from a fertilizing egg injected with WGA. As with BAPTA, the luminescence change still occurred in the presence of WGA (Fig. 6B). The time taken for a half-maximal change in the MAPK_AR_ signal was 4 h 5 min (±61 min, s.e.m.) for WGA-injected eggs (*n* = 29), which is not significantly shorter than control fertilizing eggs. This result suggests that the ongoing presence of Ca^2+^ oscillations has rather little influence upon the start of MAPK inactivation.

In mouse eggs, the inhibition of MPF activity with roscovitine leads to a decrease in MAPK activity in the absence of a Ca^2+^ increase (Phillips et al., 2002; Rogers et al., 2006). Thus, another way of studying the influence of Ca^2+^ signals upon the time course of MAPK inactivation during fertilization is to determine the effects of roscovitine. Fig. 6C,D shows that roscovitine addition to MAPK_AR_- or MAPK_AR_Δ4-injected mouse eggs caused a change in luminescence that resembles that seen in fertilizing eggs. Roscovitine caused egg activation as indicated by the formation of pronuclei, but it did not cause a significant Ca^2+^ increase under these conditions (supplementary material Fig. S4). The delay from the addition of roscovitine to the start, and to the half maximal change in signal, in three eggs was ∼6 h for the MAPK_AR_ signal but was hard to define due to the lack of initial basal luminescence with MAPK_AR_. The delay to the start of the MAPK_AR_Δ4 signal was 3 h 35 min (±81 min; *n* = 12, s.e.m.) after addition of roscovitine. The response to roscovitine was more variable than at fertilization, but within the same range of times. These data, therefore, suggest that the change in MAPK_AR_ and MAPK_AR_Δ4 signals after roscovitine addition was similar to that seen after fertilization, and is clearly much more delayed compared with the addition of U0126 (Fig. 6C,D compared to Fig. 2B,C). These data suggest that although Ca^2+^ oscillations might have some small effect upon the timing of MAPK inactivation, a decline in MPF activity alone is sufficient to trigger the same dynamics of MAPK inactivation as seen at fertilization.

### The role of phosphatases in the decline in MAPK

**Fig. 7.**
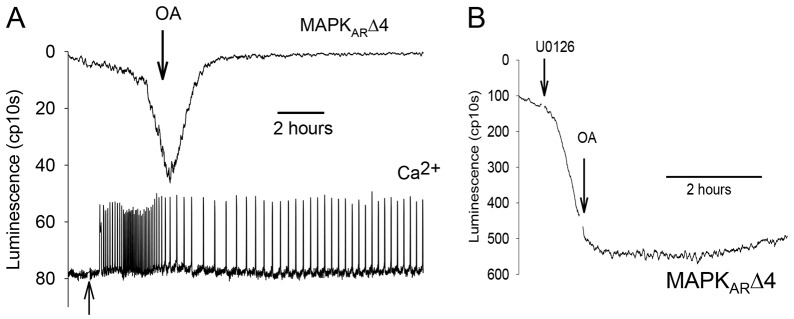
**Effect of okadaic acid on eggs undergoing a decrease in MAPK activity.** Conditions for fertilization were the same as Fig. 2, and eggs were expressing MAPK_AR_Δ4. (A) A representative trace from one of 11 eggs that was undergoing Ca^2+^ oscillations and had started to show a change in MAPK_AR_Δ4 luminescence. Okadaic acid (OA) 1 µM, was added at the arrow. Sperm were added to the dish at the point indicated by the lower arrow. (B) A representative trace from one of 15 eggs expressing MAPK_AR_Δ4 where 50 µM U0126 was added first exactly in the manner of Fig. 1C, except there was a later addition of okadaic acid (1 µM) towards the end of the change in MAPK_AR_Δ4 signal.

The protein phosphatase inhibitor okadaic acid can prevent the decrease in MAPK activity at fertilization and, hence, prevent the formation of pronuclei (Moos et al., 1995). We wanted to investigate the role of an okadaic-acid-sensitive phosphatase in the dynamics of MAPK inactivation. However, okadaic-acid-sensitive phosphatases, such as PP2A, play a role in meiotic arrest and early application of okadaic acid can itself cause a decline in MPF levels (Chang et al., 2011). To avoid interference in these early events of egg activation, we added okadaic acid after Ca^2+^ oscillations had proceeded for >1 h (after MPF will have declined). In Fig. 7A, after the MAPK_AR_Δ4 signal had started to change, we found that there was change in luminescence, which reverted back to unfertilized levels. There was also a persistence of Ca^2+^ oscillations after adding okadaic acid, which is probably caused by the fact that okadaic acid prevents pronuclear formation, which blocks PLCζ nuclear localization (Larman et al., 2004). Similar results were seen when the MAPK_AR_ probe was applied to fertilizing eggs (see supplementary material Fig. S5). These data suggest that okadaic acid can prevent a decrease in ERK1 activity after the initiation of the decline in ERK1 signaling. Because ERK1 phosphorylation is restored after okadaic acid application, this implies that a kinase activity is still able to phosphorylate ERK1 even when it is normally in the process of being dephosphorylated. MEK is clearly the crucial kinase required for restoring ERK1 activity as shown by experiments on unfertilized eggs. As shown previously (Fig. 2), the addition of the MEK inhibitor U0126 caused a relatively rapid change in MAPK_AR_Δ4 signal in unfertilized eggs. The subsequent addition of okadaic acid did not affect the U0126-derived MAPK_AR_Δ4 signal change, meaning that MAPK activity is not restored when MEK is inactivated. Taken together, these data show MEK is still active at the onset of ERK1 inactivation.

## DISCUSSION

A decrease in MAPK activity is a common feature of egg activation at fertilization in animals (Dupré et al., 2011; Marangos et al., 2003, Dumollard et al., 2011). A reduction in MAPK activity in mammalian eggs is essential for the progression into interphase, marked by the formation of pronuclei (Moos et al.,1995; Moos et al., 1996). Despite its significance in completing egg activation, much less is known about how the decrease in MAPK is controlled compared with our knowledge about the decrease in MPF (CDK1–cyclin-B) activity at fertilization (Ducibella and Fissore, 2008). Here, we have used luciferase-based reporters to examine the specific changes in Mos levels and MAPK activity in living eggs during fertilization. To our knowledge, this is the first study in any species to measure the dynamics of MAPK inactivation in synchrony with Ca^2+^ oscillations in eggs at fertilization. In addition, this is the first study to examine whether Mos destruction occurs during activation at fertilization and to test whether Mos plays a role in MAPK inactivation in mammalian eggs.

### The use of a split luciferase reporter of MAPK

A major problem in studying MAPK in mammalian eggs is the limited material that is available to carry out precise biochemical assays of activity. A FRET-based indicator for ERK activity has been reported (Harvey et al., 2008). However, when we used this in mouse eggs, we found that the small FRET change with this reporter made it unsuitable for the long-term recordings that our experiments require. In order to monitor the activation state of ERK1, we used a split-luciferase chemiluminescent reporter strategy based upon a click beetle luciferase. Our split-luciferase reporter relies upon a conformational change associated with dimerization of ERK1 or ERK2 that has been shown to occur in response in double phosphorylation of ERK (Cobb and Goldsmith, 2000). Using the split luciferase, we found a large change in luminescence (∼10-fold), which is characteristic of split-luciferase reporters (Kim et al., 2007; Zhang et al., 2007). The large change in luminescence we detect with our MAPK probes appears to be linked to a phosphorylation-induced conformational change and not dimerization as such. One of our probes, MAPK_AR_Δ4, uses an ERK1 sequence that is defective in dimerization, and yet it still shows a 10-fold change in signal. Because luciferase complementation effectively detects molecular movement it appears that some conformation change is occurring within our MAPK probes in response to phosphorylation.

There are a number of probes that consist of a single molecule with the N- and C-termini of luciferase at each end, and a pair of nested proteins that bind to each other (Kim et al., 2007). In some cases, interaction of the two internal components leads to an increase in luciferase luminescence (Kim et al., 2007), whereas with other reporters there is a luminescence decrease (Zhang et al., 2007). Our MAPK_AR_ reporter constructs, like endogenous ERK1/2, are phosphorylated in unfertilized eggs, and after blocking MEK with U0126 they are dephosphorylated, again like the endogenous ERK1/2. At the same time, a marked increase in luminescence is detected. Hence, the MAPK_AR_ reporters show an inverse relationship between phosphorylation (ERK interaction) and luminescence (i.e. complementation of the N- and C-terminal luciferase domains). Consequently, our probes cannot be interpreted as directly monitoring ‘dimerization’ of ERK1 subunits given that one assumes this should involve subunits coming together and lead to an increase in luminescence. The importance of phosphorylation for the change in luminescence is shown by the observation that a mutant MAPK_AR_, which lacks the T202 and Y204 phosphorylation sites (Khokhlatchev et al., 1998), did not show any change in luminescence with either U0126 application or at fertilization (Fig. 2; supplementary material Fig. S2). These data show that ERK1 phosphorylation and activation are reported effectively by our MAPK_AR_ proteins.

We also found that the MAPK_AR_Δ4 reporter showed a higher basal luminescence level than the wild-type MAPK_AR_ reporter. This suggests that phosphorylation can still trigger a conformation change between the two ERK1 protomers. We can assume that there is likely to be a greater degree of complementation between the split domains of luciferase with the MAPK_AR_Δ4 reporter. Notably, it has been previously shown that the Δ4-ERK1 protein can be dephosphorylated at the same rate as wild-type ERK1 (Lidke et al., 2010), so it is equally valid as a reporter of ERK1 dephosphorylation for our studies of MAPK inactivation. For our experiments, the MAPK_AR_Δ4 protein was valuable because the higher basal luminescence meant that it was easier to determine the time at which the change in MAPK activity occurred during egg activation at fertilization. As well as the current use in mouse eggs, our MAPK probes might be valuable for studies in other oocyte and eggs where MAPK activity changes play a key role in maturation and activation after fertilization (Dupré et al., 2011). In the sea urchin, biochemical experiments have led to a disagreement about whether MAPK activity is high or low in the unfertilized eggs, and to how it changes in response to Ca^2+^ at fertilization (Philipova and Whitaker, 1998; Carroll et al., 2000; Zhang et al., 2006). Live imaging of eggs using our probes might help resolve such issues.

### The timing of a late change in MAPK activity

In this study, we were able to monitor the dynamic changes in ERK1 inactivation alongside Ca^2+^ oscillations, during mammalian fertilization in single eggs. Ca^2+^oscillations served as the activation stimulus and a temporal marker for the onset and completion of egg activation events in mammals (Ducibella and Fissore, 2008). Previous studies have shown that the onset of MAPK inactivation occurs somewhere between second polar body formation (∼1 h after Ca^2+^ oscillations start) and the formation of pronuclei, which occurs ∼3 h later (Marangos et al., 2003). Both MAPK_AR_ and, particularly, MAPK_AR_Δ4, reported the initial MAPK decrease as being at ∼2 h after Ca^2+^ oscillations commence. MAPK inactivation is known to be a strict requirement for the pronuclei to form in mouse zygotes (Moos et al., 1996), and our data are consistent with these findings. A recent report measured the levels of phosphorylated ERK1/2 in individual fertilizing mouse eggs using immunostaining, and showed that the detectable decrease in phosphorylation occurred after pronuclear formation (Soeda et al., 2013). Thus a role of MAPK in pronuclear formation was questioned. Our data suggests that a large fraction of the MAPK decrease does indeed occur after pronuclear formation. However, the onset of MAPK inactivation is apparent before pronuclear formation, consistent with other reports measuring MAPK activity in lysates (Marangos et al., 2003). Importantly, our approach using MAPK indicators was able to bring a much higher degree of time resolution and sensitivity compared to other biochemical assays.

Despite an extended profile for the modification in MAPK activity and its initiation during Ca^2+^ oscillations, the overall MAPK change was only marginally affected by ongoing Ca^2+^ signals. Neither terminating nor extending the series of Ca^2+^ oscillations prevented the decrease in MAPK activity occurring. This is consistent with a previous study that measured MAPK using an *in vitro* kinase assay in mouse eggs injected with lectin (Marangos et al., 2003). However, we did observe a small change in the timing of the MAPK decrease by terminating Ca^2+^ oscillations early with BAPTA. This might be mediated by an indirect effect upon MPF activity, because Ca^2+^ signals are known to rapidly alter cyclin B levels (Madgwick and Jones, 2007). Our data do not support the idea that Ca^2+^ signals promote MAPK activity (Hatch and Capco, 2001). We also found that roscovitine-induced MPF activity decrease alone leads to a decrease in MAPK activity (Phillips et al., 2002). Notably, we found that the dynamics, with the distinct onset of MAPK inactivation, induced by roscovitine was similar to that seen at fertilization. Thus our data suggest that the MPF reduction specifically triggers a sequence of events that initiates the onset of MAPK inactivation.

### The role of Mos in MAPK inactivation

Previous studies in *Xenopus* and mouse oocytes have shown that Mos is the essential trigger for increased phosphorylation of both MEK and ERK1/2. This leads to the increase in MAPK activity during oocyte maturation (Verlhac et al., 1996; Dupré et al., 2011). After fertilization, the MAPK pathway has decreased activity, and the destruction of Mos has been implicated in causing the reduced MAPK activity (Dupré et al., 2011). In fertilizing ascidian oocytes the endogenous level of Mos has not been measured, but the decline in Mos levels is evidently necessary because any exogenous Mos expression prevents fertilized oocytes from progressing to interphase, leading to multiple rounds of polar body emission (Dumollard et al., 2011). Mos–Cherry expression exerts these effects at very low concentrations, which is consistent with studies in frog eggs, where Mos has been measured at 3 nM (Ferrell, 1999). The physiological levels of Mos in mouse eggs have not been reported but it is likely to be relatively low because ∼1500 mouse eggs per lane are required to detect Mos on western blots (Weber et al., 1991). We investigated the destruction of Mos in fertilizing mouse eggs using luciferase-tagged Mos, analogous to studies that monitored cyclin B destruction at fertilization using GFP. Based on our previous studies calibrating PLCζ–luciferase signals (Nomikos et al., 2011), we estimated that the Mos–luc expression in our experiments was ∼1–10 nM, which is well within the physiological range. Despite this, we found no evidence for increased destruction of Mos–luciferase at fertilization. However, Mos–luc levels started to autonomously degrade in unfertilized mouse eggs. These results are consistent with previous studies showing that endogenous Mos protein declines with time in unfertilized mouse eggs (Shoji et al., 2006; Suzuki et al., 2010). Therefore, there is no indication that fertilization causes increased Mos degradation. This refutes the idea that a decline in Mos levels is responsible for MAPK inactivation in mouse eggs.

In order to further investigate the role of Mos at fertilization, we intentionally overexpressed Mos–luc at several orders of magnitude greater concentrations than in the experiments monitoring Mos–luc destruction. This Mos–luc overexpression showed no effect in terms of pronuclear formation in mouse eggs, as judged by the cessation of Ca^2+^ oscillations. In addition, we measured MAPK activity, with MAPK_AR_, and simultaneously overexpressed Mos–Cherry in fertilized mouse eggs. Given that detecting a signal from a fluorescent protein, such as Cherry, in cells requires concentrations of several hundred nanomolar (Niswender et al., 1995), as with the Mos–luc, it appears that we are expressing exogenous Mos protein orders of magnitude above the endogenous levels in an egg. We again found that overexpression of Mos–Cherry did not have any effect on MAPK inactivation, which showed similar dynamics to that in normal fertilized mouse eggs. These data strongly suggest that Mos degradation does not play a direct role in MAPK inactivation of mouse eggs after fertilization. Interestingly, Mos expression can block mitotic cell cycles in early ascidian embryos (Dumollard et al., 2011), but Mos does not have such an effect in mouse embryos (Kashima et al., 2007). Thus, there might be mechanistic differences in the way that MAPK is regulated between urochordate and mammalian zygotes.

### MAPK inactivation is driven by a phosphatase activity

Mouse eggs can be induced to form pronuclei by treatment with U0126, which inactivates MEK and leads to a decrease in ERK1/2 activity (Phillips et al., 2002). Moreover, pronuclear formation in mouse zygotes can be prevented by overexpression of a constitutively active form of MEK. These data suggest a model of egg activation in which there is a decrease in MEK and then a decrease in ERK1 activity at fertilization. This scenario presumes a decrease in upstream components, such as Mos, as the primary cause of reduced MEK activity. Two separate lines of evidence from the current study now suggest that the anticipated upstream decline in MAPK pathway activity does not apply to activation in mouse eggs. Firstly, as discussed above, we did not detect evidence for any additional decrease in Mos levels caused by fertilization. Secondly, we found that okadaic acid fully reversed the marked MAPK decline after it had been initiated at fertilization. Okadaic acid has previously been shown to cause nuclear envelope breakdown and an increase in MAPK activity when applied to pronuclear stage mouse zygotes (Moos et al., 1995). However, this effect was only reported for pronuclear stage zygotes treated with okadaic acid for 4 h, so it was not possible to assess how rapidly and to what extent MAPK activity was restored. Our data reveals that okadaic acid induces rapid resumption of full MAPK activity, and this suggests that Mos and MEK remain active in the egg even at the end of the activation process. MEK activity might prevail even in the face of a decline in Mos because a low level of Mos appears to persist in activated eggs after pronuclear formation (Weber et al., 1991), and this might still be sufficient to maintain MEK activity. This activity retention is feasible because MAPK cascades can display hysteresis, and once activated by an upstream kinase, a very low level of subsequent stimulation is sufficient to keep the pathway active (Markevich et al., 2004).

Because the Mos–MEK pathway remains active, the simplest explanation for MAPK inactivation in mouse zygotes is that an increase in phosphatase activity drives down the MAPK activity during fertilization. Previously, it has been shown that injecting a constitutively active MEK blocks pronuclear formation in fertilized mouse eggs (Moos et al., 1996). In this case, the endogenous phosphatase activity might be insufficient to compete against exogenous MEK. This putative phosphatase activity at fertilization, which might overcome the endogenous MEK activity, would need to be maintained for a prolonged period in order to ensure no return of MAPK activity. The scenario we propose is similar to mitosis, where there is a decrease in overall phosphatase activity during entry into meiosis and then, with progression towards interphase, there is a return to a prolonged and higher level of phosphatase activity (Barr et al., 2011). The identity of the phosphatase responsible for the decline in MAPK activity remains to be elucidated, but could include either PP2A or PP1 (Smith et al., 1998). Our proposed model for the completion of activation of mouse zygotes is that a rise in a phosphatase activity alone, which is independent of Mos, drives a decrease in MAPK activity that leads to pronuclear formation.

## MATERIALS AND METHODS

### Collection and handling of gametes

Mature mouse eggs were collected from super-ovulated MF1 female mice as described previously (Saunders et al., 2002; Larman et al., 2004). Procedures on mice were carried out under the regulations of a UK Home Office Project Licence and were approved by a local Ethical Review committee. The cumulus cells were dissociated from eggs by treatment with hyaluronidase in M2 medium. Eggs were otherwise maintained at 37°C in M2 and were microinjected using pressure applied to micropipettes as described previously (Swann et al., 2009). For live recordings from eggs, the zona pellucida of mouse eggs were removed by brief treatment with acid Tyrodes solution, and the eggs were then attached to a glass slide that formed the base of a heated chamber. The chamber contained HKSOM medium with 100 µM luciferin (Larman et al., 2004; Swann et al., 2009). For luminescence experiments, eggs were equilibrated for ∼1 h in medium containing luciferin before the start of an experiment. Sperm were collected from the cauda epididymis of F1 (C57×CBA) males and capacitated in T6 medium for 2–3 h before addition to eggs. To initiate fertilization, a small volume ∼20 µl of capacitated sperm was pipetted into the drop containing the eggs. All chemicals were obtained from Sigma (Poole, Dorset UK) unless otherwise stated.

### Genetically encoded reporters

A bioluminescent reporter based upon interaction of the *Renilla* luciferase N- and C-terminal domains that results from ERK2 dimerization has previously been characterized (Kaihara and Umezawa, 2008). However, *Renilla* luciferase uses coelenterazine as a substrate. We used the click beetle luciferase to design and prepare a new ERK probe because this enzyme uses the more stable luciferin as a substrate. The click beetle luciferase emits green light and has been shown to be pH insensitive over the range of pH 6–8 (Kim et al., 2007). To make a luciferase reporter for human ERK1 phosphorylation and activity, two tandem-connected ERK1 molecules were inserted between the N-terminal (1–413) and C-terminal (395–543) regions of click beetle luciferase (see Fig. 1). A 32-residue linker comprising 16 tandem glycine-serine repeats (16GS) was inserted between the two ERK1 molecules that were amplified from a GFP–ERK1 template (http://www.addgene.org/14747/). All PCR-amplified ERK1 constructs were first inserted into the pCR®-Blunt II-TOPO® vector (Invitrogen) and then subcloned into the pCR3 vector (Invitrogen). The denoted restriction sites were used in construction of the ERK1 luciferase construct: *Bam*HI–N-Luc(1–413)-*Eco*RI–ERK1–*Eco*RI–16GS–ERK1–*XhoI-*C-Luc(395–543)–*XbaI*. The construct is referred to as the MAPK activity reporter (MAPK_AR_). A control ERK1–luciferase construct that is deficient in its ability to dimerize was prepared using the dimerization-deficient mutant ERK1Δ4(Pro193–Asp196) template which was amplified from the GFP–ERK1-Δ4 plasmid kindly provided by Phillipe Lenormand (Lidke et al., 2010). This is referred to as MAPK_AR_Δ4. In addition, a luciferase reporter containing the ERK1 double phosphorylation-site mutants (T202A, Y204F) was generated with a Quick-Change Mutagenesis kit (Stratagene) using the oligonucleotide primers 5′-GGCTTCCTGGCGGAGTTTGTGGCTACGCG-3′ and 5′-CCCTCGCCGATGTACTGCAACTGCGTGTA-3′. This is referred to as MAPK-DM. Plasmids encoding the mouse Mos (Verlhac et al., 2000) and ascidian Mos (Dumollard et al., 2011) were kind gifts of Remi Dumollard. Mouse Mos was amplified from pRN3-Mos, cloned into pCR3, and the click beetle luciferase was then inserted in-frame at the Mos C-terminus. In order to express the various expression constructs, we generated the corresponding cRNAs for microinjection in mouse eggs. *In vitro* synthesis of capped RNAs was performed using *Nde*1-linearized plasmids with the T7 mScript™ Standard mRNA Production System (Cambio) as per the manufacturer's instructions, and these RNAs were then polyadenylated as described previously (Swann et al., 2009). For antisense Mos–luc, the cRNA was made via the SP6 promoter using the AmpliCap Message Maker kit (Epicentre).

### Immunoblotting

To assess for the presence of endogenous and recombinant expression of ERK1, mouse eggs were collected in SDS buffer, heated for 5 min at 100°C and proteins were separated by SDS-PAGE (Nomikos et al., 2011). Immunoblotting was then performed as described previously (Verlhac et al., 1996). Following transfer onto polyvinylidene difluoride membrane (Immobilon-P; Millipore) using a semi-dry transfer system (Trans-Blot SD; Bio-Rad) in buffer (48 mM Tris-HCl, 39 mM glycine, 0.0375% SDS) at 22 V for 4 h and blocking overnight in 5% skimmed (low-fat) milk in TBS (10 mM Tris-HCl, pH 7.5, 140 mM NaCl) containing 0.1% Tween-20 (TBS/Tween), the membrane was incubated for 1 h with the appropriate primary antibody. ERK1 was detected using monoclonal antibody against diphosphorylated ERK1/2 (1∶1000, M9692 Sigma) and ERK1 (1∶500, G-8; sc-271269, Santa Cruz Biotechnology) antibodies. Detection of horseradish-peroxidase-coupled secondary antibody was achieved using enhanced chemiluminescence detection (ECL, Amersham Biosciences). All experiments were repeated at least three times.

### Luminescence and fluorescence measurement

In our experiments, the luminescence of eggs depicts the emission from the recombinant luciferase-tagged proteins expressed from the cRNAs that we injected. Eggs were incubated in M2 medium for 2–3 h to allow expression of the luciferase-based constructs before starting an experiment. The fluorescence recording is to detect the Ca^2+^-sensitive dye Oregon Green BAPTA dextran (OGBD) (Invitrogen, UK). This consisted of a stock of concentration of 1 mM in KCl/Hepes buffer that was then diluted 1∶1 with cRNA for a particular luciferase construct. The luminescence and fluorescence measurements were recorded from eggs using one of two intensified charge-coupled device (ICCD) photon-counting imaging systems (Photek Ltd, St Leonards on Sea, UK) that switch between recording modes and have been described previously (Campbell and Swann, 2006; Swann et al., 2009). Mos–Cherry fluorescence from eggs was measured using a 550-nm excitation and a 590-nm long-pass emission filter with a 565-nm dichroic mirror, at the start of the experiment. Then, during the rest of the experiment, a 490-nm and 520-nm filter block was used to measure OGBD fluorescence and luciferase luminescence (Campbell and Swann, 2006). Unless otherwise stated, the luminescence traces were filtered using a running average of 5 min. The fluorescence traces are all in arbitrary units because we only recorded the occurrence of Ca^2+^ oscillations and not their absolute magnitude. For *in vitro* fertilization experiments, data were only analyzed for eggs where the Ca^2+^ oscillations could be recorded from start to finish without a major deviation of the baseline, which otherwise occurred occasionally in eggs that moved during recording. Data was collected and analyzed using the Photek camera software and SigmaPlot. Further details of the imaging system and methodology has been given previously (Campbell and Swann, 2006; Swann et al., 2009). These experiments were carried out over the course of 2 years and we noted that there was some variation in the timing of Ca^2+^ oscillations seen in control fertilizations, and so key experiments compared interventions with groups of eggs studied within the same month as control eggs.

## Supplementary Material

Supplementary Material
