# Supplementary material for: The dynamics of MAPK inactivation at fertilization in mouse eggs
Source: J Cell Sci. 2014 Jun 15;127(12):2749–60. doi: 10.1242/jcs.145045 (PMC4058113; doi:10.1242/jcs.145045)
Supplement: Supplementary Material [file supp_127_12_2749__index.html]

The dynamics of MAPK inactivation at fertilization in mouse eggs — Supplementary Material 

# The dynamics of MAPK inactivation at fertilization in mouse eggs

## JCS145045 Supplementary Material

**Files in this Data Supplement:**

- **Supplementary Material**
