# Supplementary material for: The dynamics of MAPK inactivation at fertilization in mouse eggs
Source: J Cell Sci. 2014 Jun 15;127(12):2749–60. doi: 10.1242/jcs.145045 (PMC4058113; doi:10.1242/jcs.145045)

Supplementary Figure Legends.

Supplementary 1. Western blots of mouse eggs using the MAPK antibody. In A, lane 1 used 50 control eggs, and lane 2 used 50 eggs that were injected with $MAPK_{AR}$ cRNA and incubated for 4hr. In B a similar experiment is shown where lane 1 shows a blot of 50 control eggs, and then two there two more separate groups of 50 eggs that were injected with $MAPK_{AR}$ cRNA and then either treated (+) or not treated with (-) U0126. The methods are as described in Fig.2. The relative levels of $MAPK_{AR}$ are much less than the endogenous ERK1/2 which can be seen as doublet in both blots.

Supplementary 2. Luminescence from an egg expressing $MAPK_{AR}\Delta4$, but that did not fertilize. Shown is one of 7 eggs that had been injected with $MAPK_{AR}\Delta4$ RNA and OGBD. The conditions are otherwise the same as in Fig 3. There are no Ca^{2+} oscillations and the luminescence recording from $MAPK_{AR}\Delta4$ shows only a gradual drift in signal with time.

Supplementary 3. MAPK-DM control trace and fertilization. In both cases the conditions were otherwise the same as in Fig.3 with recording of luminescence from MAPK-DM (that cannot be phosphorylated) and fluorescence from OGBD. In A is shown an example of one of 12 control eggs where no sperm were added to eggs and no Ca^{2+} oscillations occurred. In B an example is shown of one of 14 eggs where sperm were added (at the arrow) and Ca^{2+} oscillations occurred. The luminescence of MAPK-DM did not show the marked (~10 fold change) characteristic of $MAPK_{AR}$ and $MAPK_{AR}\Delta4$.

Supplementary 4. Activation of eggs by roscovitine. In A there is a trace of one of 5 eggs following the addition of roscovitine (50μM), with the luminescence of $MAPK_{AR}\Delta4$ and the fluorescence of OBGD to measure Ca^{2+}. The conditions were the same as Fig 6 except that measurements of Ca^{2+} were made and showed no substantial Ca^{2+} increase in response to roscovitine. B shows an image of some eggs activated by roscovitine that have formed single pronuclei compared to eggs that formed two pronuclei after fertilization. The presence of pronuclei is most evident by the nucleoli in the appropriate focal plane. The fertilization experiment was at the end of a ~15 hours recording.

Supplementary 5. The effect of okadaic acid on eggs undergoing a decrease in MAPK. The conditions are the same as in Fig.7 except that the $MAPK_{AR}$ probe was used. The example shown is one of 13 eggs that underwent Ca^{2+} oscillations at fertilization, and then once the $MAPK_{AR}$ signal has started to change, 50μM okadaic acid was added (OA at the arrow). After a delay the $MAPK_{AR}$ luminescence returns to pre-fertilization levels and the Ca^{2+} oscillations return.

Supplementary 1. Western blots of $MAPK_{AR}$ injected eggs.

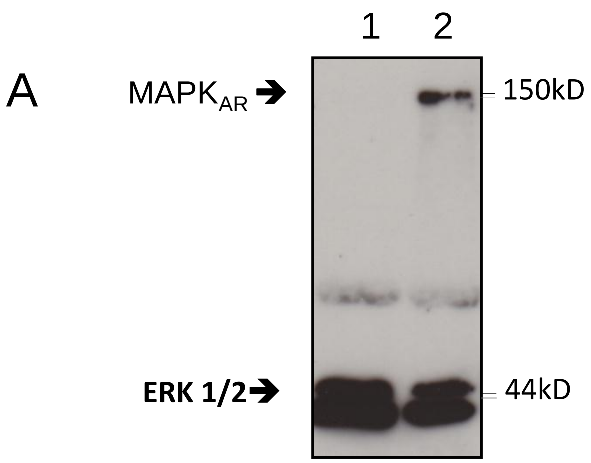

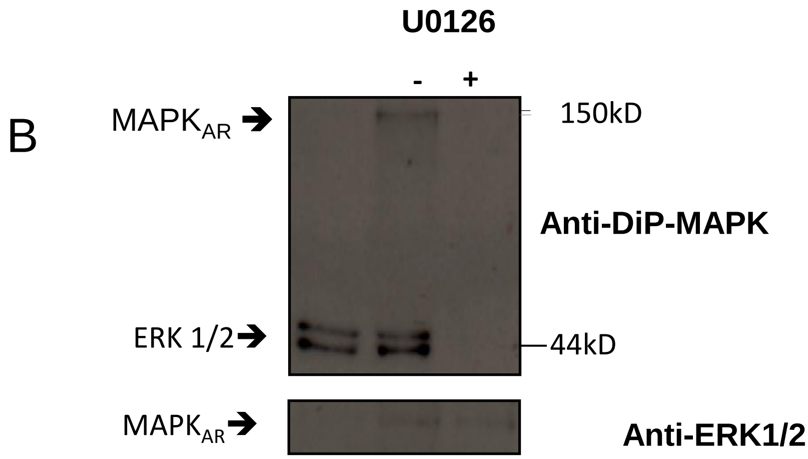

Supplementary 2. $MAPK_{AR}\Delta4$ control traces without fertilization

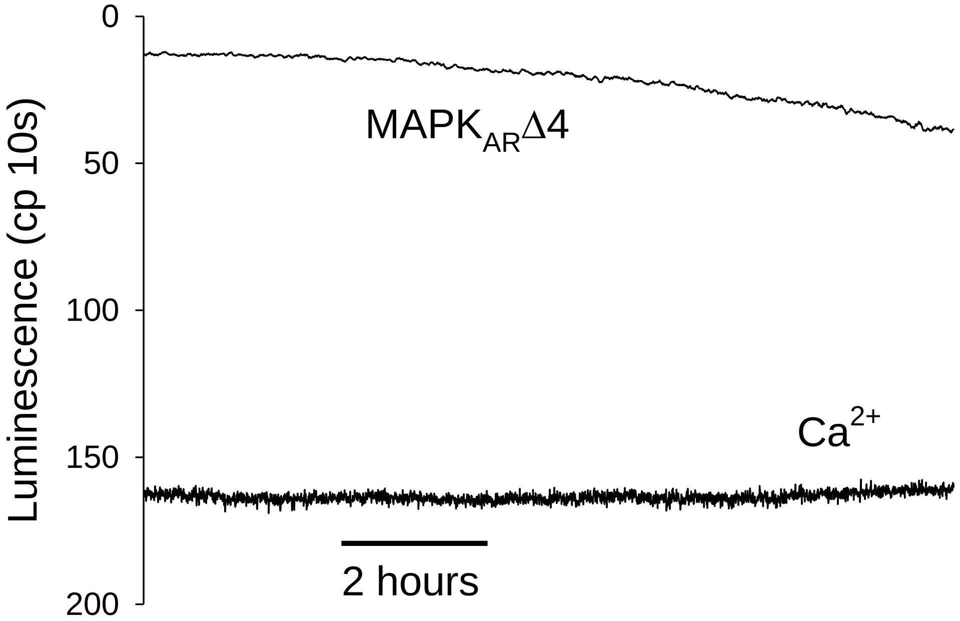

Supplementary 3. MAPK-DM control trace and fertilization.

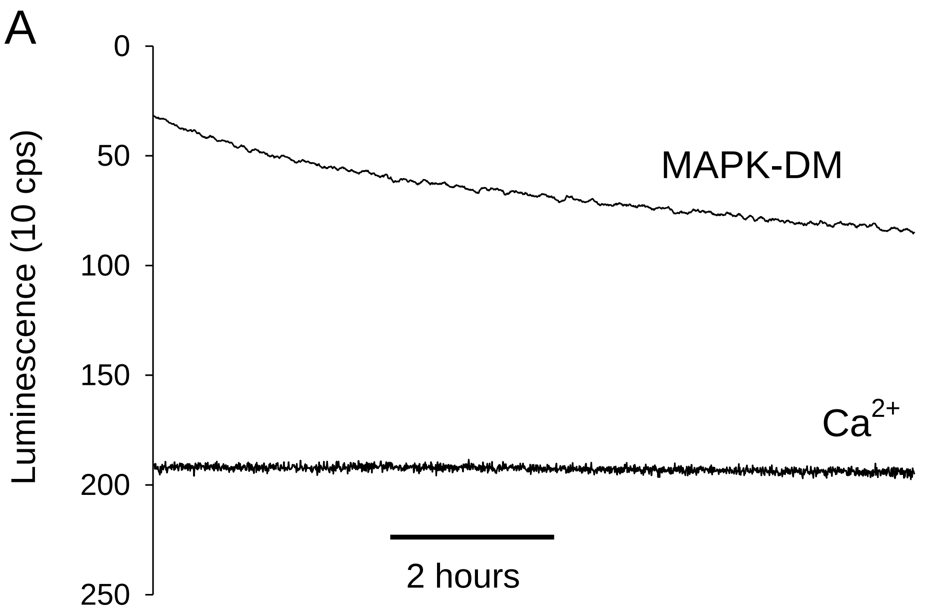

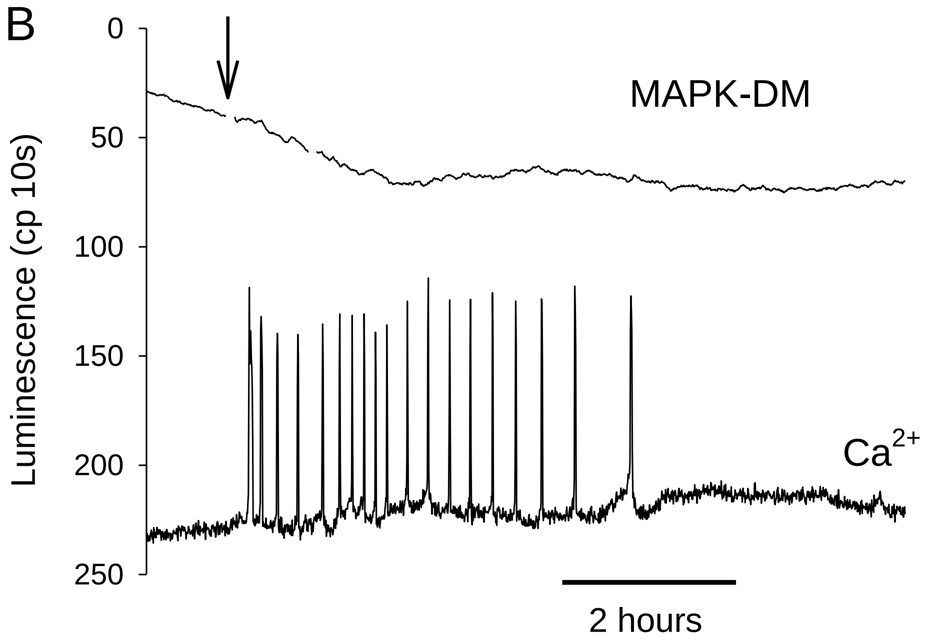

Supplementary 4. Activation of eggs by roscovitine.

A

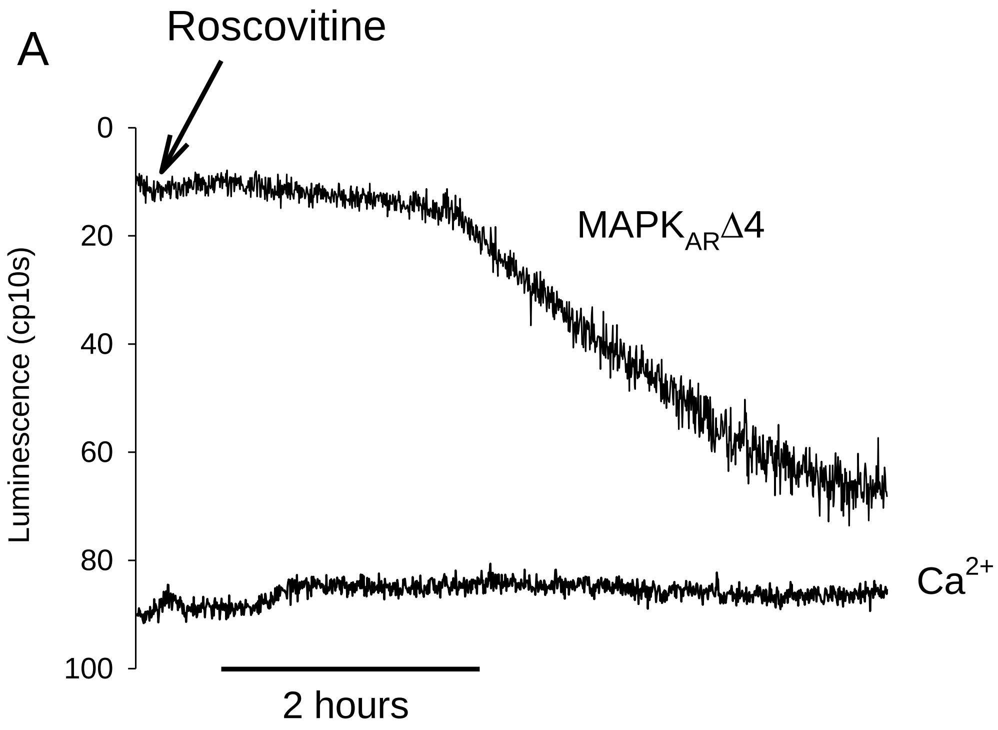

B

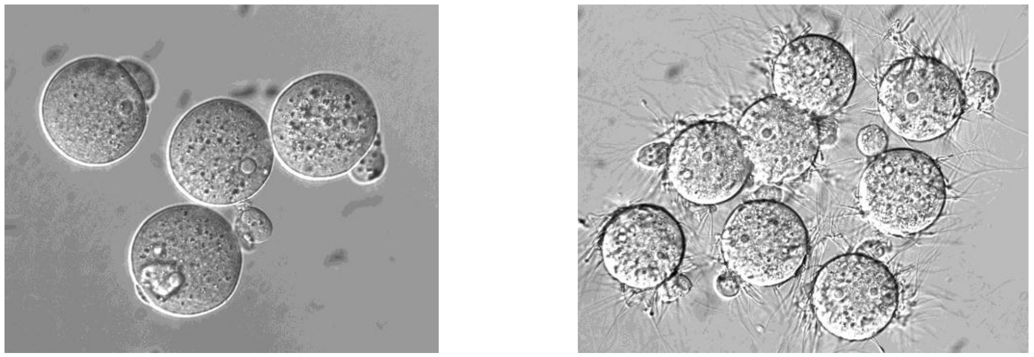

Roscovitine

Fertilization

Supplementary 5.
The effect of okadaic acid on eggs undergoing a decrease in MAPK

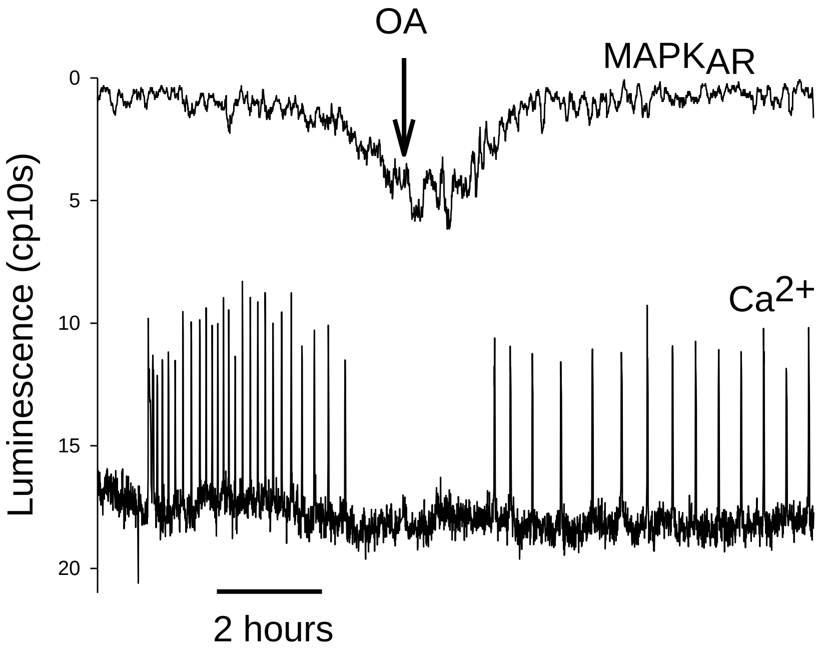

Supplement: Supplementary Material [file supp_127.12.2749_JCS145045.pdf]
